# Allelochemicals and Signaling Chemicals in Plants

**DOI:** 10.3390/molecules24152737

**Published:** 2019-07-27

**Authors:** Chui-Hua Kong, Tran Dang Xuan, Tran Dang Khanh, Hoang-Dung Tran, Nguyen Thanh Trung

**Affiliations:** 1 College of Resources and Environmental Sciences, China Agricultural University, Beijing 100193, China; 2 Graduate School for International Development and Cooperation, Hiroshima University, Hiroshima 739-8529, Japan; 3 Agricultural Genetics Institute, Pham Van Dong Street, Hanoi 122000, Vietnam; 4 Center for Expert, Vietnam National University of Agriculture, Hanoi 131000, Vietnam; 5 Faculty of Biotechnology, Nguyen Tat Thanh University, Ho Chi Minh 72820, Vietnam; 6 Institute of Research and Development, Duy Tan University, Da Nang 550000, Vietnam

**Keywords:** allelopathy, plant neighbor detection, signaling interactions, chemical defense, belowground chemical interactions, pest management, sustainable agriculture, non-targeted chemical analysis

## Abstract

Plants abound with active ingredients. Among these natural constituents, allelochemicals and signaling chemicals that are released into the environments play important roles in regulating the interactions between plants and other organisms. Allelochemicals participate in the defense of plants against microbial attack, herbivore predation, and/or competition with other plants, most notably in allelopathy, which affects the establishment of competing plants. Allelochemicals could be leads for new pesticide discovery efforts. Signaling chemicals are involved in plant neighbor detection or pest identification, and they induce the production and release of plant defensive metabolites. Through the signaling chemicals, plants can either detect or identify competitors, herbivores, or pathogens, and respond by increasing defensive metabolites levels, providing an advantage for their own growth. The plant-organism interactions that are mediated by allelochemicals and signaling chemicals take place both aboveground and belowground. In the case of aboveground interactions, mediated air-borne chemicals are well established. Belowground interactions, particularly in the context of soil-borne chemicals driving signaling interactions, are largely unknown, due to the complexity of plant-soil interactions. The lack of effective and reliable methods of identification and clarification their mode of actions is one of the greatest challenges with soil-borne allelochemicals and signaling chemicals. Recent developments in methodological strategies aim at the quality, quantity, and spatiotemporal dynamics of soil-borne chemicals. This review outlines recent research regarding plant-derived allelochemicals and signaling chemicals, as well as their roles in agricultural pest management. The effort represents a mechanistically exhaustive view of plant-organism interactions that are mediated by allelochemicals and signaling chemicals and provides more realistic insights into potential implications and applications in sustainable agriculture.

## 1. Introduction

Once germinated, plants establish their locations in permanent places, which include various biotic and abiotic factors during the whole growth stages. Far from being passive in the face of environmental factors, particularly in the case of stressors, sedentary plants use many elegant strategies to increase their chances of survival. Some of these strategies involve the production and release of specific metabolites [1,2]. Plants produce and release a wide variety of primary and secondary metabolites, including carbohydrates, proteins, vitamins, and other organic chemicals with low molecular weight. Nearly 5–30% of all photosynthetically fixed carbon is transferred to the environments through the release of metabolites [3]. Over 80% of secondary metabolites or natural products identified have been derived from plants so far. Therefore, plants represent an important source of active ingredients with multiple functions for the environment, as well as human affairs [4,5]. Importantly, the active ingredients from plants participate in the defense of plants against microbial attack, herbivore predation, and/or competition from other plants.

Plants have developed response systems to suppress other plant competitors and they defend against herbivores and pathogens. These systems include constitutive defense and inducible defense [6,7]. Both constitutive-and inducible-defense involve primary and secondary metabolites from plants. However, secondary metabolites, such as flavonoids, terpenoids, alkaloids, and cyanogenic glycosides, are primarily involved in the inducible defense mechanism [8]. These defensive metabolites play important roles in plants and other organism interactions. In particular, plants may affect the performance of neighboring plants via allelopathy. Allelopathy is an interference mechanism, in which plants produce and release defensive metabolites (i.e., allelochemicals), exerting a negative effect on conspecific and heterospecific plants [9]. Allelochemicals can have substantial impact on the growth and establishment of neighboring plants, while the neighboring plants influence the plant defensive biochemistry, altering the production of allelochemicals [10]. Besides repelling herbivores, exerting direct antimicrobial properties and suppressing plant competitors [11], plant-derived chemicals can act as alarm signals to warn neighboring plants of an imminent herbivorous or pathogen attack [12,13], or serve as an inter-and intra-plant signal for the detection of neighbors, even including kin and non-kin individuals within a species [14,15,16]. These signaling chemicals mediate plant sensing and communication [16]. Through the production and release of allelochemicals and signaling chemicals, plants suppress competing plant species and act against herbivores and attacking microbes, in particular, regulate the soil microbial community and encouraging beneficial symbioses and alter the belowground ecological interactions [17,18]. Accordingly, plant-derived allelochemicals and signaling chemicals have been attracting attention as practical materials for agricultural pest management. An in-depth understanding of implication and application of plant-derived allelochemicals and signaling chemicals can develop novel ecological strategies for sustainable agriculture.

## 2. Allelochemicals

Plants may interfere with the establishment and growth of neighboring plants through competition, allelopathy, or both. Differing from competition for resources, allelopathy involves the release of allelochemicals from living or dead plants into the environment [9]. Accordingly, the identification of allelochemicals from plants and their environments is key to understanding the plant-plant allelopathic interactions. So far, numerous allelochemicals have been investigated and identified from a variety of plant species. These allelochemicals are chemically diverse, being represented by phenolic compounds (simple phenolics, flavonoids, coumarins, and quinones), terpenoids (monoterpenes, sesquiterpenes, diterpenes, triterpenes, and steroids), alkaloids and nitrogen-containing chemicals (non-protein amino acids, benzoxazinoids, cyanogenic glycosides), and many other chemical families [19].

### 2.1. Allelochemicals from Herbaceaous and Woody Species

**Figure 1:**
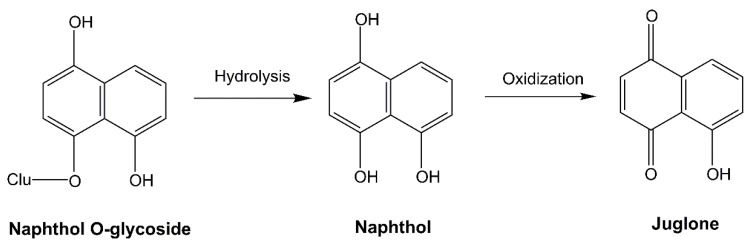
The first allelochemical juglone originated from black walnut.

The first verified allelochemical was juglone, a 1,4-naphthoquinone, which originates from black walnut (*Juglans nigra*) [20]. Black walnut is a woody species that interferes with understory plants. This allelopathic phenomenon was recorded thousands of years ago, but the allelochemical juglone was only isolated and identified several decades ago [20,21]. In black walnut, juglone occurs in a non-toxic naphthol O-glycoside form. The naphthol O-glycoside biosynthesized in living tissues may be released from leaves, barks, and roots to the environment. The naphthol O-glycoside is rapidly transformed into aglycone, a less phytotoxic naphthol, by hydrolysis or soil microbial interactions, and then oxidized to phytotoxic juglone [22]. The phytotoxic juglone is formed from non-toxic naphthol O-glycoside by environment interactions once released (Figure 1).

The classical example of allelochemical juglone indicates that the dynamics of allelochemicals and their fate in the environment are crucial factors for allelopathic interactions. The action of allelochemicals requires their presence at the phytotoxic level in the vicinity of the target plants. Once released from plant tissues, allelochemicals have to interact with abiotic and biotic factors. The majority of allelochemicals finally endure a series of soil processes, such as retention, transport, and transformation [23,24]. Particularly, allelochemicals interact with soil microorganisms at rates of significance. Allelochemicals that are released from root systems are able to exert an effect on soil microorganisms, while soil microorganisms consume and decompose allelochemicals. Thus, soil microorganisms are an important determinant of allelochemical action [25,26].

**Figure 2:**
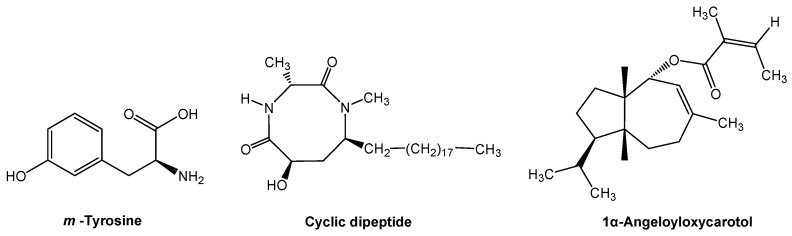
Novel allelochemicals from fine fescue grasses, Chinese fir and giant ragweed.

Many old and new allelochemicals have been investigated and discovered in the past 10 years [19]. Several novel allelochemicals have been identified from herbaceaous and woody species. Fine fescue grasses (*Festuca* spp.) are important resources for improved ecological function under rangeland stress environments. The grasses may displace the neighboring plants by releasing phytotoxic root exudates into the soil. The major allelochemical m-tyrosine (Figure 2), which is a non-protein amino acid, was identified from the phytotoxic root exudates. The non-protein amino acid is responsible for allelopathic action, through m-tyrosine, fescue grasses interfere with root development of other plants, subsequently displacing neighboring plants [27]. Several hundred non-protein amino acids have been identified in plants. The non-protein amino acids protect plants against herbivores, pathogens, and other plant competitors. Many of the non-protein amino acids are highly toxic to plants and microbes, and some act as a feeding deterrent to animal predators [28]. Furthermore, non-protein amino acids play roles in the plant, soil, and ecosystem interactions, such as nutrient acquisition, signaling, and stress response [29].

Chinese fir (*Cunninghamia lanceolata*) is an important conifer tree species for industrial wood production. However, its regeneration failure and productivity decline have become a serious problem, due to continuous monocultures [30,31]. The problem is mainly caused by a novel allelochemical cyclic dipeptide (6-hydroxy-1,3-dimethyl-8- nonadecyl-[1,4]-diazocane-2,5-diketone) that is released by Chinese fir trees themselves (Figure 2). The cyclic dipeptide is released from Chinese fir roots and it penetrates into the soil. The concentrations of this allelochemical is increased with successive monocultures, resulting in self-inhibition (i.e., intraspecific allelopathy). The allelochemical cyclic dipeptide has a highly phytotoxic activity, which limits offspring growth in Chinese fir plantations [32,33]. Interestingly, growing the Chinese fir with other tree species, such as *Michelia macclurei*, may reduce the release of allelochemical cyclic dipeptide and increased its degradation in the soil, which results in a shift from self-inhibition to facilitation [34]. The presence of a “good” neighbor, like *M. macclurei*, may decrease or dilute allelochemicals that are released from an allelopathic species into the environment. The production and release of allelochemicals depends on co-occurring plant species, and thus a plant may modify its defensive strategy based on the identity of neighbors [10,11,14]. Plant neighbor recognition and allelochemical response has important implications for plant coexistence in vegetation and community assembly.

In cropping systems, crop plants have coexisted with specific weedy species. Many weeds are allelopathic and release allelochemicals that affect crop plants. Giant ragweed (*Ambrosia trifida*) is one of the most allelopathic weeds and it usually occurs on arable lands, which reduces crop productivity [35]. 1α-Angeloyloxycarotol (Figure 2), a carotane-type sesquiterpene, has been isolated and identified from giant ragweed. The carotane-type sesquiterpene inhibits the growth of wheat at a low concentration. Furthermore, 1α-angeloyloxycarotol was detected from giant ragweed infested and residue amended soils in sufficient quantities. Therefore, 1α-angeloyloxycarotol that is released from giant ragweed into the soil could be a key allelochemical in giant ragweed infested wheat fields [36]. Phytochemicals with carotane skeletons and other terpenoids have strong bioactivity and they are one of the largest classes of active ingredients from plants. Therefore, the search for allelochemicals has been focused on this class of phytochemicals [19,37].

### 2.2. Allelochemicals from Allelopathic Crop Cultivars

**Figure 3:**
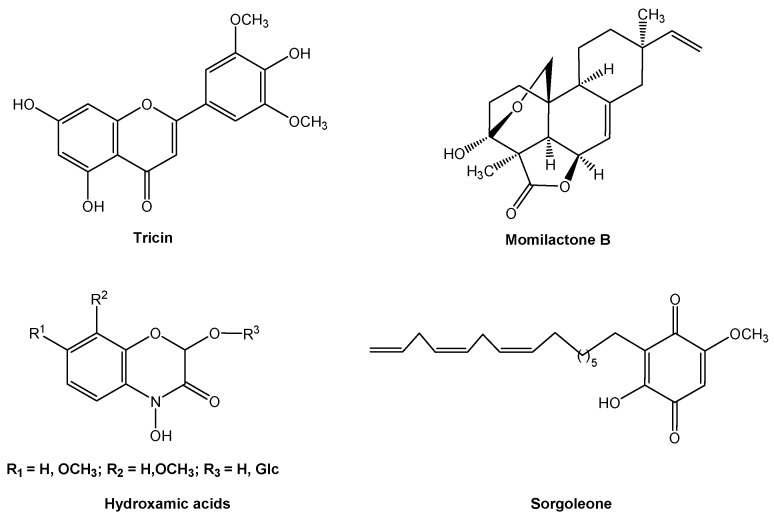
The key allelochemicals involved in the principal grain crops.

Allelopathic weeds produce and release allelochemicals that affect crop plants. Fortunately, a few crop cultivars are allelopathic as well, which results in a natural inhibition of weeds. These allelopathic crop cultivars have been investigated and identified from several germplasm collections, particularly for the principal grain crops rice and wheat [38,39,40,41,42]. Allelopathic crop cultivars can produce and release their own ‘herbicides’ (i.e., allelochemicals) to suppress weeds, permitting ecological weed control. Therefore, much effort has gone into investigating and determining allelochemicals from allelopathic crop cultivars. Thus far, the key allelochemicals from allelopathic cultivars of the principal grain crops have been identified, such as tricin and momilactone B from rice (*Oryza sativa*) [43,44], benzoxazinoid hydroxamic acids from wheat (*Triticum aestivum*) and maize (*Zea mays*) [45], and sorgoleone from sorghum (*Sorghum bicolor*) (Figure 3) [46].

**Figure 4:**
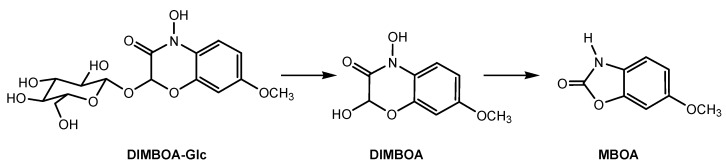
2-(2,4-dihydroxy-7-methoxy-1,4-benzoxazin-3-one)-β-D-glucopyranose (DIMBOA-Glc) and its degradation products in soil.

Tricin (5,7,4′-trihydroxy-3′,5′-dimethoxyflavone) and momilactone B are important allelochemicals that have been discovered from allelopathic rice cultivars and many plant species [43,47]. In particular, allelopathic rice seedlings biosynthesize 5,4′-dihydroxy-3′,5′-dimethoxy-7-O-β- glucopyranosyl-flavone and 7,4′-dihydroxy-3′,5′-dimethoxy-5-O-β-glucopyranosyl-flavone. Two flavone O-glycosides are exuded from allelopathic rice roots to the environment and are then transformed into their aglycone form, 5,7,4′-trihydroxy-3′,5′-dimethoxyflavone (i.e., tricin) to exert allelopathic effects on paddy weeds and soil microorganisms [48]. Similarly, allelopathic wheat seedlings biosynthesize 2-(2,4-dihydroxy-7-methoxy-1,4-benzoxazin-3-one)-β-D-glucopyranose (DIMBOA-Glc), which is stored in vacuoles. When leached from the roots to the soil, DIMBOA-Glc is rapidly transformed to its aglycone 2,4-dihydroxy-7-methoxy-1,4-benzoxazin-3-one (DIMBOA) and then 6-methoxy-benzoxazolin-2-one (MBOA) (Figure 4) [49,50]. DIMBOA and MBOA are the dominant allelochemicals in wheat-weed allelopathic actions. They can effectively suppress weeds that reassociated with wheat crop. MBOA has relatively broad-spectrum bioactivity when compared with DIMBOA, and it is more resistant toward degradation in soil [45,49].

Allelochemical DIMBOA belongs to benzoxazinoid hydroxamic acids that were derived from 2-hydroxy-2H-1,4-benzoxazin-3(4H)-one. Besides wheat, other cereals (maize and rye, but not rice and sorghum) also produce benzoxazinoids that contain the 2-hydroxy-2H-1,4-benzoxazin- 3(4H)-one skeleton. The involvement of benzoxazinoids in the defense of plants against various organisms has been investigated during the past decades. Benzoxazinoid concentrations in cereals have a correlation with fungi pathogenic resistance [51,52]. DIMBOA and MBOA modified the soil microbial community structure to their advantage through a change in fungi populations in cereal rhizosphere [53]. In particular, benzoxazinoids that are released by roots of cereals may shape the rhizosphere microbiota, decrease plant growth, increase plant defense, and suppress herbivore performance in the next plant generation. Subsequently, root-secreted DIMBOA and MBOA drive plant-soil feedbacks on growth and defense by modifying root-associated microbial communities [54]. A further clarification of the interactions between benzoxazinoids and soil microbes, particularly for soil-borne pathogenic fungi, may offer potential application for disease management in cropping systems.

Sorghum is an important cereal in the semiarid tropics. Sorgoleone, 2-hydroxy-5-methoxy-3- [(8′Z,11′Z)-8′,11′,14′-pentadecatriene]-p-benzoquinone (Figure 3), is one major component of sorghum root exudates. The root exudates of sorghum generally contain 85–90% pure sorgoleone with considerable variation among genotypes [46,55]. Sorgoleone has been characterized as an allelochemical to suppress many weeds. Sorgoleone has a greater activity than that of classical allelochemical juglone and many phenolics and terpenoids [56]. Sorgoleone strongly inhibits hydroxyphenylpyruvate dioxygenase (HPPD), which interferes with photosynthesis [57]. In particular, sorgoleone is not immediately degraded in the presence of soil microflora, and has a residual effect on soil [58]. After sorgoleone was formulated as a wettable powder, the weed-suppression of formulated sorgoleone is more effective than that of sorgoleone itself with crop plants that are tolerant to the powder [56].

In conclusion, the exploitation of allelochemicals may be of particular value for agricultural pest management. For this purpose, tricin, momilactone B, benzoxazinoid hydroxamic acids, and sorgoleone from the principal grain crops that are described above are the most extensively studied allelochemicals. Together, these efforts represent an exhaustive view of allelopathic crops against pests through their own pesticides (i.e., allelochemicals) in cropping systems.

## 3. Signaling Chemicals

Plants’ chemical defenses include constitutive and inducible systems. In this context, plant defensive metabolites are constitutively a dynamic and their production and release can be induced by biotic and abiotic factors. Inducible defensive responses in plants are known to be locally and systemically triggered by signaling chemicals. Biotic-induced plant defenses have been mainly studied in conjunction with insect or pathogen attack [6,59]. An increasing number of studies have revealed that competing neighbors influence plant defensive biochemistry, altering the production of allelochemicals. It is well-known that allelopathic crop plants can increase their allelochemical production in the presence of weeds [60,61,62,63]. It appears from the results that allelopathic crop plants may detect the presence of weeds and respond by increasing allelochemical levels. In plant–plant coexistence systems, a plant first may detect and recognize its neighbors, and then initiate allelopathic action to regulate inter-specific or intra-specific interactions. Plant neighbor detection and allelochemical response are two inseparable processes that occur when one more plants interact [14,64]. There is a wealth of information regarding allelochemicals in plant-plant allelopathic interactions. However, there is a lack of data when it comes to plant-derived signaling chemicals. Therefore, much effort has focused on the investigation and identification of plant-derived signaling chemicals in the recent years.

### 3.1. Air-Borne Siganling Chemicals

**Figure 5:**
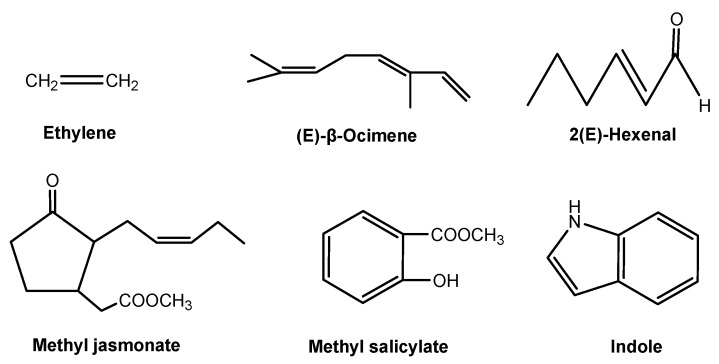
Plant-derived airborne signaling chemicals.

Plant and other organisms signaling interactions take place both aboveground and belowground. Plants may detect or identify neighboring organisms through plant volatiles as air-borne signals [2,65], while root exudates drive belowground signaling interactions [17,66]. Aboveground signaling interactions that are mediated by air-borne signaling chemicals are well established. However, soil-borne signaling chemicals in plant-organism belowground interactions are largely unknown. Therefore, plant-derived signaling chemicals have mainly focused on air-borne chemical signals, including ethylene, methyl jasmonate and salicylate, indole, and several volatile terpenoids (Figure 5).

Ethylene is the first gaseous plant hormone and it acts as an air-borne signaling chemical within the plant in response to its plant neighbors, herbivores, pathogens, or other attackers. However, plants release a wide array of air-borne signaling chemicals in response to herbivores and pathogens. Two of the best studied air-borne signaling chemicals in plants are methyl jasmonate and salicylate (Figure 5). Jasmonic acid and salicylic acid are common secondary metabolites in plants. Non-volatile jasmonic acid and salicylic acid may be methylated to produce volatile methyl jasmonate and salicylate once the plants are attacked or infested [67]. In general, methyl jasmonate is a signal that defends plants against herbivores, while methyl salicylate is responsible for the resistance response of plant to microbes [68]. Similar to methyl jasmonate and salicylate, several volatile terpenoids that are emitted from damaged or attacked leaves serve as a signaling chemical to induce defenses in neighboring plants. Among them, (E)-β-ocimene and 2(E)-hexenal (Figure 5) are two typical signaling chemicals of volatile terpenoids. In response to the attack, infested leaves release (E)-β-ocimene to increase the resistance of un-infested leaves, inducing the expression of defense-related genes in neighboring un-infected leaves [65]. 2(E)-Hexenal is an odor component of various fruits, which is produced in response to bacterial pathogenesis. This chemical as a widespread volatile signal to induce defense responses in plants, particularly for the accumulation of sesquiterpenoid phytoalexins, as was described many years ago [2]. A recent study shows that indole is a herbivore-induced air-borne signaling chemical. Herbivore-induced indole enhances the induction of defensive chemicals in neighboring plants. Therefore, indole is a reliable and effective air-borne signaling chemical that prepares systemic tissues and neighboring plants for incoming attacks [69].

Responses to the biotic factors in a plant’s life may be anticipated by signaling chemicals from neighboring plants. In particular, plants seem to eavesdrop on signaling chemicals that are released from neighbors and respond by tailoring their defensive strategies in order to enhance their own fitness [65]. Air-borne signaling chemicals may directly elicit the production of defensive metabolites. Alternatively, signaling chemicals exposure may allow for nearby plants to ready their defenses. In this manner, plants may avoid investing fitness limiting resources in the production of costly defenses before attackers arrive. Furthermore, air-borne signaling chemicals may guide host location and host selection by parasitic plants. The parasitic plant *Cuscuta pentagona* seedlings can distinguish the air-borne signaling chemicals from host tomato and non-host wheat and preferentially grow toward the host tomato plants. Several terpenes, such as β-phellandrene and β-myrcene, may be responsible for this effect, indicating that directed growth of aboveground plants could also be induced by air-borne chemical cues [70].

### 3.2. Soil-Borne Signaling Chemicals

**Figure 6:**
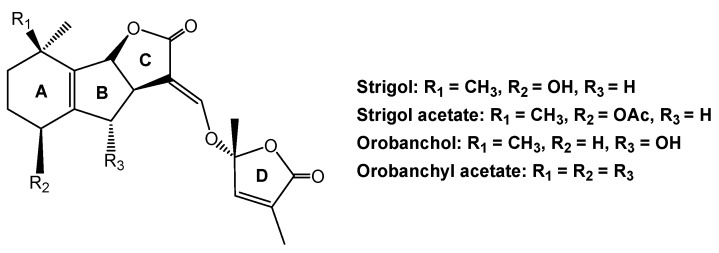
Strigol derivatives that are produced by the roots of host and non-host plants.

Parasitic plants also use soil-borne signaling chemicals for host location. The roles of host-derived signaling chemicals in belowground host location by parasitic plants have been previously determined. Host root-secreted signaling chemicals influence seed germination and the growth of parasitic plants that attach to host roots, and induce haustorial development by these parasites [71,72]. Strigol is the first seed germination stimulant for parasitic witchweed (Striga lutea) that was discovered in several decades ago [73]. Subsequently, several strigol derivatives, including orobanchol and orobanchyl acetate, are regarded as signaling chemicals between parasitic plants, such as *Striga* Spp., *Orobanche* spp., and their hosts [74,75]. These chemicals contain a four-ring backbone structure (Figure 6), which was later classified as a group of plant hormones that are known as strigolactones. Strigolactones may be produced by the roots of host or non-host and then exuded into the rhizosphere [72]. In recent decades, new strigolactones and strigolactone-like chemicals have been identified from the root exudates of many plant species, such as sorghum, red clover (*Trifolium pratense*), and several medicinal herbs [76].

**Figure 7:**
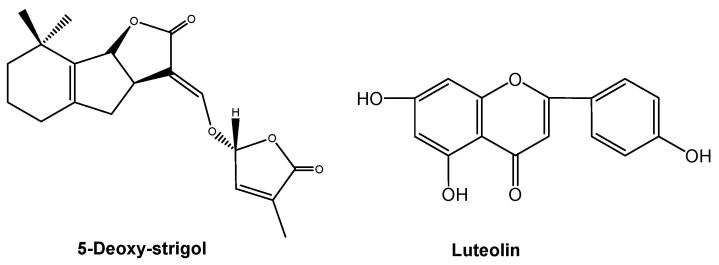
5-Deoxy-strigol and luteolin involving in signaling interactions between plants and their symbiotic mycorrhizal fungi or rhizobia.

Root-secreted strigolactones stimulate seed germination in plants that parasitize the roots of other plants. Interestingly, they also act as signaling chemicals for symbiotic interactions with the arbuscular mycorrhizal fungi that colonize roots and facilitate the uptake of soil nutrients by plants. 5-Deoxy-strigol (Figure 7) is a key signaling chemical for fungal symbionts and parasitic plants in roots [77]. Root-secreted signaling chemicals mediate plant-microbe interactions. Phenolic flavones may be chemical signals that participate in plant-microbe signaling interactions. A typical flavone, luteolin (Figure 7), was reported as a signaling chemical in Fabaceae plants and symbiotic rhizobia 20 years ago [78]. Mycorrhizal fungi and rhizobia both promote root growth, and subsequently shoot growth. Therefore, strigolactones and flavones that are involved in the regulation of mycorrhizal fungi and rhizobia play important roles in coordinating plant growth belowground and aboveground.

**Figure 8:**
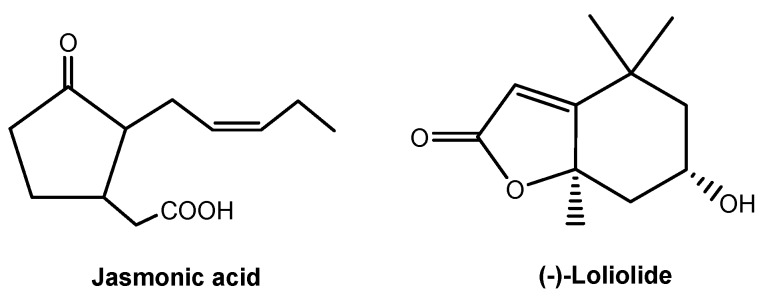
Soil-borne signaling chemicals in plant-organism belowground interactions.

Root-secreted strigolactones and flavones mediate belowground signaling interactions among plant species or plants with symbiotic microbes. However, strigolactones and flavones do not appear to have a universal role in belowground signaling interactions, and thus there may be other soil-borne signaling chemicals awaiting identification. Jasmonic acid (Figure 8) is a ubiquitous signaling chemical that elicits the production of defensive metabolites in plants [79,80,81]. A few studies have documented root-secreted jasmonic acid and its roles in belowground signaling interactions [64,80]. A recent study shows that the most ubiquitous lactone, (-)-loliolide (Figure 8), is involved in belowground signaling interactions. Allelopathic wheat plants can detect and recognize the root-secreted (-)-loliolide from their interacting plant species and then respond by increased allelochemical DIMBOA. More importantly, (-)-loliolide was found in more than 100 plant species and their rhizosphere soils. (-)-Loliolide has good soil mobility and it appears to be a soil-borne signaling chemical for plant recognition and allelochemical response in belowground interactions, being potentially common to all species [16]. Similarly, (-)-loliolide stimulates accumulation of defensive metabolites that are involved in plant against pathogens and herbivores [82,83]. Therefore, (-)-loliolide may be a ubiquitous, soil-borne signaling chemical in belowground ecological interactions between plants and other organisms. The regulatory network of jasmonic acid in plant defenses has been well established [80,81,84]. However, the regulatory network of (-)-loliolide as a signaling chemical in plant defenses has not yet been established. The discovery of (-)-loliolide as a common soil-borne signaling chemical, as well as a further understanding of its potential mechanisms in belowground signaling interactions, should lead to new insights into plant chemical defense strategies.

Plants have evolved elaborate chemical communication systems in response to a varying environment. However, research regarding plant-derived signaling chemicals in plant-insect signaling, plant-microbe signaling, and plant-plant signaling interactions are typically studied separately, despite their necessary linkage in nature. In particular, the induction of defense-related responses arising from insects and pathogens stressors or plant signaling involves secondary metabolites that have multiple functions, and thus plant chemical defense against the pests may be altered. Under this scenario, plant-derived signaling chemicals may be the key regulators in a network of biochemical communications for plant defense. The signaling chemicals or chemical elicitors identified and their importance for plants and other organisms’ interactions are able to confer exciting implications and applications in natural and managed ecosystems.

## 4. Roles in Sustainable Agriculture

The heavy use of pesticides for agricultural pest management is of great concern in the environment and human health. In this regard, there needs to be an alternative to such an over-reliance on pesticides in agriculture. Exploiting the potentials of plant self-defense in cropping systems is one promising option, particularly for the exploitation of allelochemicals and signaling chemicals in plants. Plant-derived allelochemicals and signaling chemicals permit ecological pest management in crop production and reduce the impact of pesticides on the environment [85,86]. Plant-derived allelochemicals and signaling chemicals thus offer an environmentally friendly alternative for agrochemicals in sustainable agriculture.

### 4.1. Allelochemical-Based Herbicide Discovery and Signaling Chemicals as Plant Elicitors

**Figure 9:**
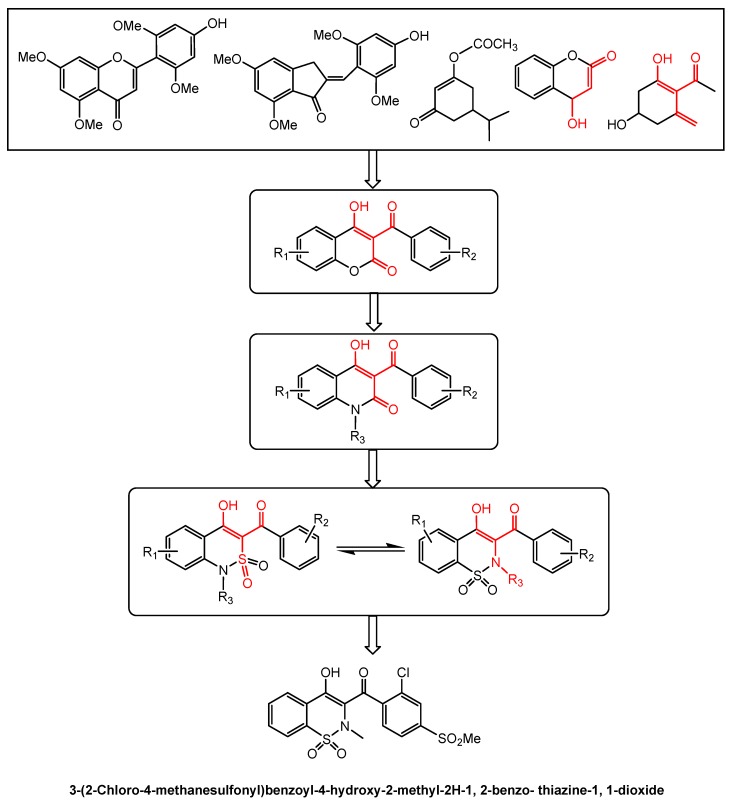
Scheme of design for an allelochemical-based benzothiazine derivative.

Allelochemicals, as new herbicide discovery efforts, may be of particular value for weed management in cropping systems [87,88,89,90]. Several allelochemicals, such as sorgoleone and tricin, have been synthesized and further modified in the past decades [87,91,92,93]. In recent years, the importance of allelochemical tricin for an allelochemical-based herbicide discovery has received a great deal of attention [94,95,96]. Tricin may be synthesized by aldol condensation of α-chloro-2-hydroxy acetophenone with aromatic aldehyde in aqueous alcoholic solution. Unexpectedly, this synthesis approach primarily yielded aurone, an isomer of tricin. This isomer had much stronger activity on weeds and pathogens than tricin itself [94,95], which indicated that the aurone isomer is an effective molecule for new herbicide discovery. In this context, there has been much effort in designing aurone-based herbicides. A series of aurone-derived compounds, including substituted aurones and benzothiazine derivatives, have been synthesized [95,96,97]. These derivatives showed highly strong bioactivities on weeds. In particular, several (2-benzoylethen-1-ol)-containing 1, 2-benzothiazine derivatives had good pre-emergent activities against weeds and inhibited HPPD interfering with photosynthesis. In addition, 3-(2-chloro-4-methanesulfonyl)-benzoyl-4-hydroxy-2-methyl-2H-1, 2-benzo-thiazine-1, 1-dioxide (Figure 9) completely inhibited the germination and growth of barnyardgrass, even at very low concentrations [96,97]. Therefore, the benzothiazine derivative was identified as a target compound for an allelochemical-based herbicide discovery effort.

After this target, benzothiazine derivative was applied into the paddy fields at the optimal doses and dominant weeds were effectively suppressed, while the rice was not negatively affected [98]. The findings suggest that the benzothiazine derivative can be recommended to provide satisfactory control of paddy weeds. In addition, the benzothiazine derivative also effectively suppresses various weeds occurring in wheat, maize, and soybean fields [98]. However, the benzothiazine derivative has different crop selectivities, an excellent selectivity for maize occurred, but was not safe for soybean [98]. Besides crop selectivity, allelochemical-based herbicides are required to have low toxicity and environmental safety. It is required to evaluate the ecological safety of the allelochemical-based benzothiazine derivative before it may be commercially used. The results obtained from toxicity tests show that the benzothiazine derivative had no toxic effects on aquatic zebrafish, soil earthworms, and soil microorganisms [98]. Therefore, the benzothiazine derivative, i.e., 3-(2-chloro-4-methanesulfonyl)-benzoyl-4-hydroxy-2-methyl-2H-1, 2-benzo-thiazine- 1, 1-dioxide (Figure 9), is an environmentally friendly allelochemical-based herbicide.

Similar to allelochemicals, plant-derived signaling chemicals have acted as plant elicitors or activator to initiate plant defense. Plant-derived signaling chemicals may be used to protect plants against herbivores, pathogens, and weeds through the induction and activation of self-defense mechanisms without exhibiting direct insecticidal, antimicrobial, or herbicidal activities. Several natural or synthesized chemicals have been identified as plant activator or plant-activator, like chemicals [99,100,101]. Some of these chemicals have been used to develop commercial products to induce resistance for use in agriculture, such as Vacciplant Fruits et Légumes (Goëmar, France) and Fytosave (Andermatt Biocontrol, Switzerland), based on the elicitors laminarin and chitosan [102,103]. Although the development of plant-derived allelochemicals and signaling chemicals has made considerable progress, they may be profitably exploited in many ways, particularly for traditional intercropping approaches and the breeding of commercially acceptable allelopathic crop cultivars.

### 4.2. Traditional Intercropping Approaches

Traditional intercropping approaches with allelopathic plants has been advocated for integrated pest management in several agro-ecosystems. *Ageratum conyzoides* is an allelopathic weed present in tropical and subtropical regions. Its allelopathic strategy has been utilized in traditional intercropping systems. In South China and Vietnam, *A. conyzoides* is often intercropped as an understory species in citrus orchards for pest control. *A. conyzoides* release allelochemicals (ageratochromene and three flavones) into soils to effectively suppress other weeds and major soil pathogens in citrus orchards [104]. In addition, *A. conyzoides* plants release volatile signaling chemicals (α-bisabolene, E-β-farnesene and β-caryophyllene) into the air to attract predatory mites (*Amblyseius* spp.) and to maintain their population density. Predatory mites are most effective natural predators of the pest citrus red mite (*Panonychus citri*). Subsequently, the intercropping of *A. conyzoides* made the citrus orchard environment more favourable for the predatory mites, resulting in a marked decrease of the citrus red mite population density at non-injurious levels [105]. Therefore, there are natural chemical mechanisms for regulating plant-organism interactions for sustainable pest control in the citrus orchard with intercropping *A. conyzoides*.

Similar natural chemical mechanisms have been successful in mixed-species tree systems. Managed tree plantations with monocultures frequently experience replant problems, which may be caused by allelochemicals from monoculture trees, as in the case of the conifer Chinese fir, which is an important timber tree species. However, productivity decline and regeneration failure of replanted Chinese fir plantations caused by allelochemicals has been a serious problem. Interestingly, the problem can be improved by mixed-species with a non-N fixing broadleaf tree species *M. macclurei*. The mixed-species *M. macclurei* alters the consequences of the belowground ecological interactions to enhance the autotoxic Chinese fir growth. In particular, the release of allelochemical of self-inhibition was reduced and its soil degradation was increased by *M. macclurei*. In this context, a natural chemical mechanism in the mixed-species plantations mediates the belowground ecological interactions [34]. Such a mechanism would be useful in the development of certain techniques to overcome or minimize the replant problem in managed tree plantations.

### 4.3. Breeding of Allelopathic Crop Cultivars

Breeding is another strategy for improving the capacity for plant self-defense against pests. The development of commercially acceptable pest resistant or defensive crop cultivars is highly desirable, and plants can be bred for increased weed suppressive activities in allelopathic crop cultivars. The development of commercially acceptable allelopathic crop cultivars has been conducted in China and Sweden. Several allelopathic rice and wheat cultivars have been developed based on breeding programs that were established in the past decade [42,106]. In 2009, the first commercially acceptable allelopathic rice cultivar Huagan-3 was released in China and several other allelopathic rice cultivars have been released in recent years. These allelopathic rice cultivars have been successfully cultivated in South China [85]. The substantial success of breeding commercially acceptable allelopathic crop cultivars and incorporating them into an integrated weed management strategy should be a promising alternative for over reliance on herbicides in cropping systems. Therefore, much effort has focused on the use of allelopathic rice cultivars as an efficient component of integrated weed management in China and United States (US) paddy fields [107,108].

Breeding for allelopathic crop cultivars have the potential for high-quality and high-yield crops with weed suppression. The weed-suppressive capacity of allelopathic crop cultivars is beneficial for crop production. However, the production and release of allelochemicals from allelopathic crop cultivars could alter crop allocation strategy, resulting in a shift from growth to defense. Accordingly, any increase in the weed-suppressive capacity by breeding is likely to impair grain yield and thus the yield of most allelopathic crop cultivars struggle to meet the commercial standards in the local crop industry. The same problem occurs in breeding for disease resistant cultivars [109]. Although the weed-suppressive capacity of allelopathic crop cultivar is a polygenic trait that is only slightly correlated with yield or other agronomic features [85,110], it is still unclear whether high-quality and high-yielding crops can be selected in parallel with strong allelopathic effects on weeds. Actually, improvements in grain yields are based on trade-offs between competitive ability and productive ability. The cooperation among kin individuals within a plant species may reduce the growth of competitive organs for increasing reproduction allocation. Accordingly, kin recognition and plant cooperation in crop species is predicted to increase grain yield by reducing the competitive effects. Actually, kin recognition among allelopathic crop cultivars does not have the constraint of high quality and high yielding. A recent study discovers allelopathic rice cultivars with the ability of kin recognition. The grain yields of these allelopathic rice cultivars were increased in the presence of kin cultivars [15]. Kin recognition of crop cultivar mixtures could be a new approach to improve the yield of the principal grain crops. Such an improvement of grain yield by kin recognition may contribute to solving the lower yield problem in the breeding of commercially acceptable pest resistant crop cultivars.

## 5. Challenge and Opportunity

The importance of plant-derived allelochemicals and signaling chemicals, as well as their roles in agricultural pest management, have received increasing attention in recent decades. Nevertheless, the plant-organism chemical interactions are a multidisciplinary science. The key of this science is the identification and detection of allelochemicals and signaling chemicals from plants and their environments. Furthermore, it requires deep knowledge of the structural characteristics of allelochemicals to facilitate the design of new pesticides. However, aspects of chemistry are overlooked, because most scientists in this field are not chemists. Differing from general phytochemicals, the collection and determination of allelochemicals and signaling chemicals require conducting in vivo, in situ, and real time from the intact and living plants [111,112]. Therefore, it remains a problem as to how to trap and identify the allelochemicals and signaling chemicals in plants and their environments.

Allelochemicals and signaling chemicals in plants and their environments are usually determined by means of gas or liquid chromatography coupled with tandem mass spectrometry (GC-MS/MS, LC-MS/MS). The GC-MS/MS is a valuable instrument for determining the constituents of mixtures, particularly for volatile chemicals. Much of the research into plant-organism chemical interactions has dealt with volatiles. This focus is primarily driven by the availability of reliable GC-MS/MS techniques. However, most allelochemicals and signaling chemicals produced and released from plants are not volatile. Moreover, analytical samples for GC-MS/MS have to be conducted at a high temperature. Therefore, GC-MS/MS is not appropriate for determining fragile and non-volatile allelochemicals and signaling chemicals that are involved in plant-organism chemical interactions. The majority of studies investigating non-volatile allelochemicals and signaling chemicals is based on LC-MS/MS analysis. LC-MS/MS has high resolution selectivity and it covers various chemicals, even with a high molecular weight. In particular, the LC-MS/MS technique is compatible with aqueous samples. However, using LC-MS/MS analysis is a mostly targeted approach, due to a lack of reliable database with chemical structures. Therefore, targeted analysis only allows for detecting chemical changes in the exudation patterns of known chemicals rather than the identification of unknown chemicals in plants and their environments. The identification of unknown chemicals requires the individual chemical components to be isolated from plant and soil samples. A bioassay-guided fractionation approach is usually is used to identify which individual components could be responsible for the self-defense or signaling interactions [14,113]. Finally, the individual of unknown chemicals isolated from plants and their environments can be identified one by one with spectroscopic analyses, particularly for MS and nuclear magnetic resonance (NMR) [114]. Such non-targeted analyses may investigate which chemicals occur in plants and their root exudates as well as the growing soils. Consequently, increasing studies are developing and applying the non-targeted chemical analysis for plant and soil samples that are involved in plant-organism belowground interactions [115,116].

One of the greatest challenges in plant-organism belowground interactions is the lack of effective and reliable methods for accurate information regarding the quality, quantity, and spatiotemporal dynamics of allelochemicals and signaling chemicals from plants and their environments. The analytical puzzle results from the complexity of the samples. The collection of allelochemicals and signaling chemicals in plant root exudates and soils is often performed under arbitrary conditions, rather than collection from the living plants in situ. The design of analytical methods requires a more realistic study of the actual field situations of a plant-soil system than the examination of under artificial conditions, particularly in the context of soil-borne allelochemicals and signaling chemicals driven belowground chemical interactions [117]. Much effort has been directed towards developing analytical methods for the examination of root-secreted chemicals that do not damage detectable root systems or affect the viability of root-associated microbiota in plant-soil systems [116,118,119].

Recent developments in methodological strategies are aiming at the quality, quantity, and spatiotemporal dynamics of soil-borne allelochemicals and signaling chemicals. Several new approaches strive to demonstrate actual quantities of the root-secreted chemicals in soils in situ [120]. Phillips et al. designed an experimental system to trap root exudates from intact tree roots in situ. This system may account for the spatiotemporal dynamics of chemicals in the root exudates and forest soils [121]. Weidenhamer et al. constructed sampling devices by means of silicone tubing microextraction. This method is feasible for monitoring the release of allelochemicals from plant roots to a soil environment [122]. One promising approach involves exploiting a microdialysis-based analytical system for dynamic sampling and quantifying chemicals from soil-grown plants [116,123]. Using a microdialysis probe that was located in soil microsites, the dynamic variations of root-secreted chemicals in plant-soil systems may be continuously monitored and identified. These positive progresses in analytical techniques are warranted to further understand the roles and functions of soil-borne allelochemicals and signaling chemicals in the plant-organism belowground interactions.

Accurate information of soil-borne allelochemicals and signaling chemicals in plants and their environments is vital in understanding the belowground chemical interactions and processes [116]. To date, it still is difficult to unambiguously identify and quantify soil-borne allelochemicals and signaling chemicals due to the complexity of plant-soil interactions [124,125,126]. In fact, there has never been a perfect method for accurate information of soil-borne allelochemicals and signaling chemicals in plant-organism belowground interactions. However, the newly-developed methods can provide a more finely-resolved picture of allelochemical and signaling chemical in plants and their environments if appropriately applied and interpreted.

Research regarding plant-derived allelochemicals and signaling chemicals has made considerable progress in theory and practice. Plant may detect or identify competitors, herbivores, or pathogens through signaling chemicals and then defend them by allelochemicals. The plant-organism interactions that are mediated by allelochemicals and signaling chemicals have important implications in natural and managed ecosystems, particularly for their importance and consequences in agro-ecosystems. The development of agricultural pest management is striving towards a future of sustainable agriculture. Plant-derived allelochemicals and signaling chemicals may regulate crop-pest interactions to improve crop production. Once herbivores, pathogens, or weeds stress crop plants, crop plants may identify and detect them and then produce and release defensive allelochemicals against the pests, providing an advantage for their own growth. Such natural chemical-defense techniques for agricultural pest management are most easily transferable to the small-farm intensive agricultural systems, particularly in organic crop-farming systems. This significant progress will reduce heavy use of pesticides, generating tremendously rewarding changes for present crop production systems. Some allelochemicals, such as momilactones A and B, also showed strong correlation with drought and salinity [127,128] and submergence [129] tolerance. Thus, the role of allelochemicals and signaling chemicals in crops should be further investigated.
